# Anti-Inflammatory Effects of Catalpalactone Isolated from *Catalpa ovata* in LPS-Induced RAW264.7 Cells

**DOI:** 10.3390/molecules24071236

**Published:** 2019-03-29

**Authors:** Hyo-Young Kim, Ah-Reum Han, Yun-Seo Kil, Eun Kyoung Seo, Chang Hyun Jin

**Affiliations:** 1 Advanced Radiation Technology Institute, Korea Atomic Energy Research Institute, Jeongeup 56212, Korea; khy5012@hanmail.net (H.-Y.K.); arhan@kaeri.re.kr (A.-R.H.); 2 College of Pharmacy, Graduate School of Pharmaceutical Science, Ewha Womans University, Seoul 03760, Korea; k_yunseo@naver.com

**Keywords:** *Catalpa ovata*, catalpalactone, lipopolysaccharide, NF-κB, IRF3

## Abstract

*Catalpa ovata* (Bignoniaceae) is widely distributed throughout Korea, China, and Japan. This study investigated the anti-inflammatory effects of catalpalactone isolated from *C. ovata* in lipopolysaccharide (LPS)-induced RAW264.7 cells. Catalpalactone significantly inhibited nitric oxide (NO) production and inducible NO synthase (iNOS) expression in LPS-induced RAW264.7 cells. The levels of cytokines such as interleukin-6 and tumor necrosis factor-α were reduced under catalpalactone exposure in LPS-induced RAW264.7 cells. Additionally, catalpalactone suppressed signal transducer and activator of transcription 1 (STAT-1) protein expression and interferon-β (IFN-β) production. Treatment with catalpalactone prevented interferon regulatory factor 3 (IRF3) and nuclear factor-κB (NF-κB) activation. Taken together, these results suggest that the anti-inflammatory effects of catalpalactone are associated with the suppression of NO production and iNOS expression through the inhibition of IRF3, NF-κB, and IFN-β/STAT-1 activation.

## 1. Introduction

Inflammation is a protective immune response that occurs when tissues are damaged by infection. Tissue damage is accompanied by pathological responses such as pyrexia, edema, and pain [1]. Lipopolysaccharide (LPS) stimulates macrophages and promotes the secretion of numerous pro-inflammatory cytokines as well as nitric oxide (NO) [2]. NO is a short-lived free radical that is synthesized by NO synthase (NOS), which exists as three isoforms: endothelial, neuronal, and inducible NOS (iNOS) [3]. In particular, iNOS is expressed in response to LPS or pro-inflammatory cytokines, and excessive NO production during the inflammatory response leads to inflammatory disorders [4,5].

LPS is recognized by toll-like receptor 4 (TLR4), and it promotes the activation of two different signal pathways: myeloid differentiation factor (MyD88)- and toll/IL-1R domain-containing adaptor inducing interferon-β (TRIF)-dependent pathways [6]. Stimulated MyD88 activates transforming growth factor β-activated protein kinase 1 and induces an inflammatory response via the nuclear translocation of nuclear factor-κB (NF-κB) [7,8]. Activated TRIF activates interferon regulatory factor 3 (IRF3) and stimulates interferon-β (IFN-β) expression [9]. IFN-β activates signal transducer and activator of transcription 1 (STAT1) and induces the expression of iNOS and inflammatory cytokines [10]. Therefore, the effective regulation of these inflammatory mediators may represent important strategies in the development of natural anti-inflammatory drugs.

*Catalpa ovata* G. Don. (Bignoniaceae) is an ornamental tree that is widely distributed across Korea, China, and Japan [11]. Previous phytochemical investigations of *C. ovata* indicated the presence of phthalides [12], naphthoquinones [13], iridoids [14], monoterpene glycosides [15], and phenolic compounds [16]. Physiological activity studies discovered that the methanol extract of the stem bark inhibits tumor necrosis factor-α (TNF-α) and NO production in RAW264.7 cells [17]. As an iridoid glycoside isolated from the stem bark, catalposide inhibited pro-inflammatory cytokine production and the transcriptional activation of NF-κB in LPS-induced RAW264.7 cells [18]. In addition, catalpalactone isolated from *C. ovata* inhibited NO production in RAW264.7 cells [19] and exerted cytoprotective effects against H_2_O_2_-induced oxidative damage in HepG2 cells [20]. However, the exact mechanism underlying the anti-inflammatory effects of catalpalactone isolated from *C. ovata* has not been reported.

Therefore, the present study examined the anti-inflammatory effects and underlying molecular mechanism of catalpalactone isolated from *C. ovata* in LPS-induced RAW264.7 cells and evaluated its potential to prevent and treat inflammatory diseases.

## 2. Results and Discussion

### 2.1. Effects of Catalpalactone on Cell Cytotoxicity

**Figure 1:**
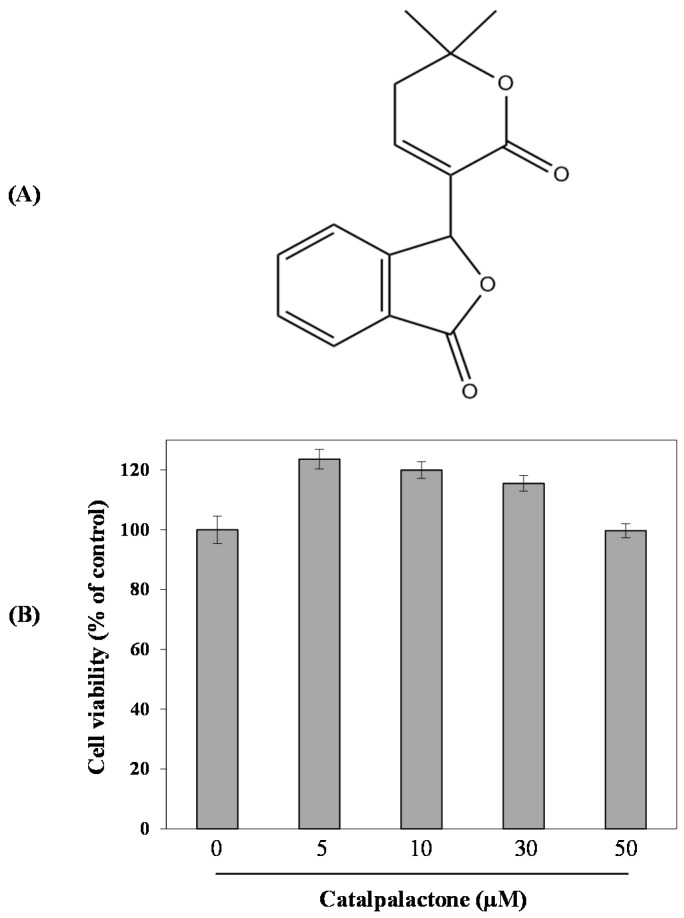
The chemical structure of catalpalactone and its effects on the viability of RAW264.7 cells. The chemical structure of catalpalactone (**A**). Cell viability was measured using an EZ-Cytox cell viability assay kit (**B**). The cells were treated with various concentrations of catalpalactone (5, 10, 30, and 50 µM) for 24 h. Data are presented as the mean ± SD (*n* = 6).

As shown in Figure 1B, catalpalactone did not exhibit cytotoxicity in RAW264.7 cells up to a concentration of 50 µM. On the basis of these results, a catalpalactone concentration of less than 50 µM was used in subsequent experiments.

### 2.2. Effects of Catalpalactone on NO Production and iNOS Expression in LPS-Stimulated RAW264.7 Cells

**Figure 2:**
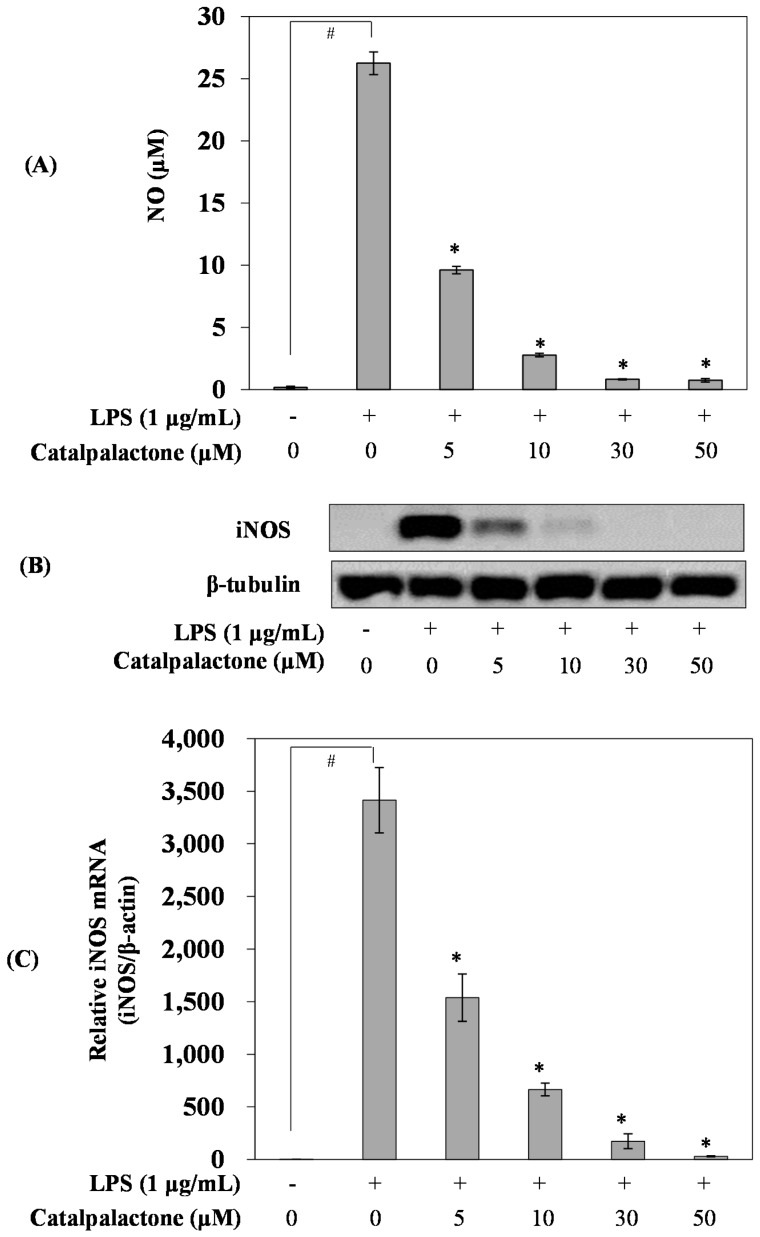
Effects of catalpalactone on nitric oxide (NO) production, and inducible NO synthase (iNOS) protein and mRNA expression in lipopolysaccharide (LPS)-stimulated RAW264.7 cells. The cells were treated with various concentrations of catalpalactone (5, 10, 30, and 50 µM) for 2 h before LPS addition (1 μg/mL) followed by incubation for 18 h (**A**). Data are presented as the mean ± SD (*n* = 6). ^#^ *p* < 0.05 vs. control cells, * *p* < 0.05 vs. LPS-treated cells. iNOS protein levels were measured via Western blotting (**B**). The cells were pretreated with various concentrations of catalpalactone (5, 10, 30, and 50 µM) for 2 h and then stimulated with or without LPS (1 μg/mL) for 18 h (**C**). iNOS mRNA expression levels were measured by quantitative real-time PCR. Data are presented as the mean ± SD (*n* = 3). ^#^ *p* < 0.05 vs. control cells, * *p* < 0.05 vs. LPS-treated cells.

NO is known to regulate physiological functions such as neurotransmission, vasorelaxation, and immune responses in a normal state [21]; however, at excessive levels, the substance damages cells and tissues, causing chronic inflammation and an autoimmune disorder [22]. LPS treatment markedly increased NO production compared with that in the control cells, whereas catalpalactone pretreatment decreased NO production in a concentration-dependent manner with an IC_50_ of 2.34 µM (Figure 2A). Park et al. reported that naphthoquinone derivatives isolated from *C. ovata* inhibited NO production, and catalpalactone had a calculated IC_50_ of 9.80 µM [19]. In line with previous findings, our results illustrated that catalpalactone strongly inhibits NO production.

Several studies have reported that NO production and iNOS expression were increased in tissues that exhibited an inflammatory response [23], and iNOS can induce NO overproduction in immunocytes stimulated by pro-inflammatory cytokines, including TNF-α and LPS [24]. To further evaluate whether the inhibition of NO production by catalpalactone was attributable to the inhibition of iNOS expression, real-time PCR and Western blotting were performed (Figure 2B,C). As expected, iNOS expression was significantly increased in the LPS-induced cells. However, catalpalactone treatment downregulated iNOS expression in a concentration-dependent manner. Recently, several studies have reported a type of naphthoquinone from *C. ovata* and *Eleutherine americana*, and it inhibited LPS-induced NO production in RAW264.7 cells [19,25]. In the present study, the naphthoquinone catalpalactone inhibited NO production by suppressing iNOS expression in LPS-induced RAW264.7 cells. Therefore, naphthoquinone compounds in *C. ovata* inhibit inflammatory activity.

### 2.3. Effects of Catalpalactone on the Production and mRNA Expression of Interleukin-6 and TNF-α in LPS-Stimulated RAW264.7 Cells

**Figure 3:**
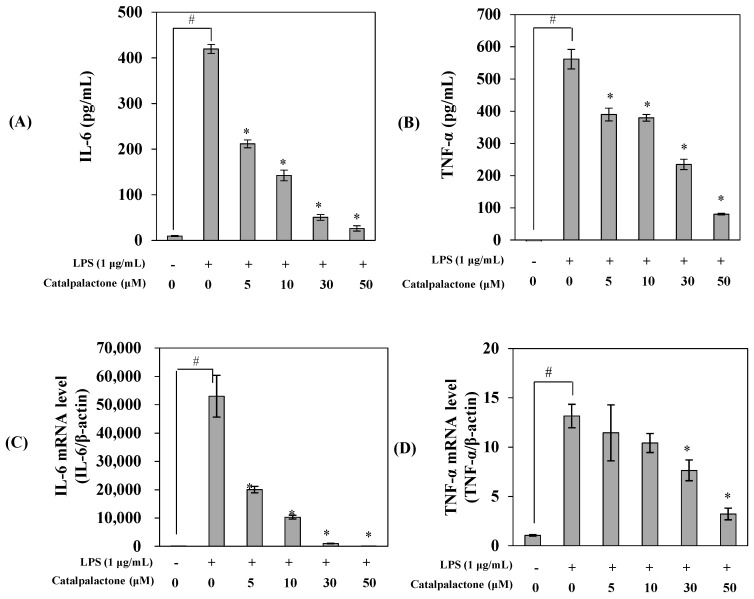
Inhibitory effects of catalpalactone on interleukin-6 (IL-6) production (**A**), tumor necrosis factor-α (TNF-α) production (**B**), IL-6 mRNA production (**C**), and tumor necrosis factor-α (TNF-α) mRNA (**D**) levels in LPS-stimulated RAW264.7 cells. The cells were pretreated with various concentrations of catalpalactone (5, 10, 30, and 50 µM) for 2 h and then stimulated with or without LPS (1 μg/mL) for 18 h. IL-6 and TNF-α production was assessed using enzyme-linked immunosorbent assay kits. IL-6 and TNF-α mRNA expression was measured via quantitative real-time PCR. Data are presented as the mean ± SD (*n* = 3). ^#^ *p* < 0.05 vs. control cells, * *p* < 0.05 vs. LPS-treated cells.

To investigate the effects of catalpalactone on the protein and mRNA expression of interleukin-6 (IL-6) and TNF-α, ELISA and real-time PCR were performed (Figure 3). LPS treatment increased the protein and mRNA expression of IL-6 and TNF-α, whereas catalpalactone treatment repressed the production and mRNA expression of these cytokines in a concentration-dependent manner. Several studies reported that catalpalactone exhibits anticancer and dopamine biosynthesis-promoting effects [13,26]. Pae et al. reported that the methanol extract of the stem bark of *C. ovata* inhibits TNF-α expression in LPS-induced RAW264.7 cells, and these effects were attributed to an iridoid [17]. Indeed, An et al. found that catalposide is a glycoside of iridoids, and it has been found to inhibit cytokine (TNF-α, IL-1β, and IL-6) expression [18]. Previous findings and our results suggest that *C. ovata* influences various physiological functions, and catalpalactone displays excellent inhibitory activity against the production of major inflammatory mediators.

### 2.4. Effects of Catalpalactone on IFN-β Production and STAT1 Activation in LPS-Stimulated RAW264.7 Cells

**Figure 4:**
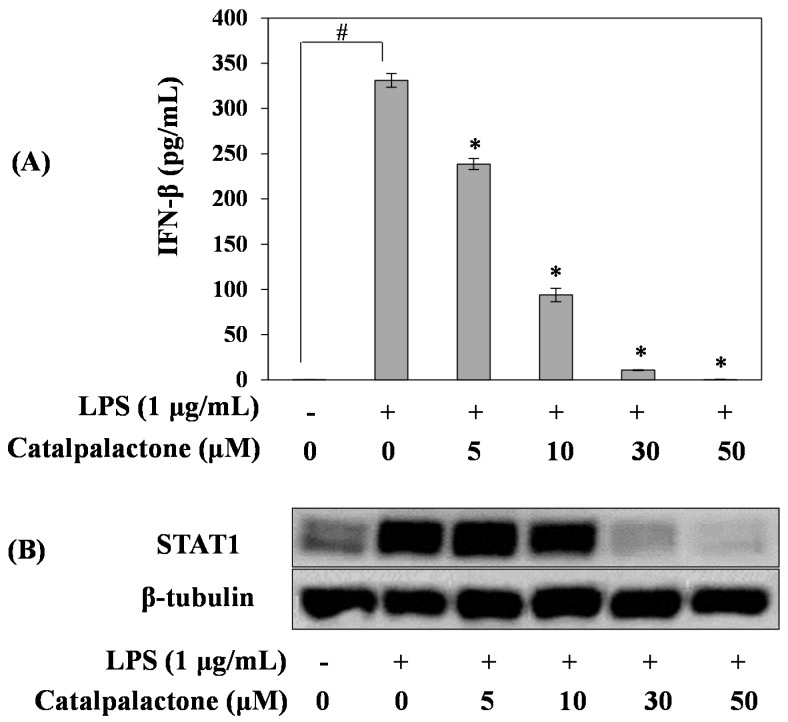
Effects of catalpalactone on interferon-β (IFN-β) production and signal transducer and activator of transcription 1 (STAT1) protein expression in LPS-stimulated RAW264.7 cells. The cells were pretreated with various concentrations of catalpalactone (5, 10, 30, and 50 µM) for 2 h and then stimulated with or without LPS (1 μg/mL) for 18 h (**A**). IFN-β production was measured using an enzyme-linked immunosorbent assay kit. Data are presented as the mean ± SD (*n* = 3). ^#^ *p* < 0.05 vs. control cells, * *p* < 0.05 vs. LPS-treated cells. STAT1 protein levels were measured via Western blotting (**B**).

To elucidate the mechanism of NO production and iNOS expression in LPS-induced RAW264.7 cells, we analyzed the effects of catalpalactone on LPS-induced IFN-β production and STAT1 activation. As shown in Figure 4A, IFN-β production in LPS-induced cells was increased compared with that in the control cells. However, catalpalactone decreased LPS-induced IFN-β production in a concentration-dependent manner. Moreover, we investigated the effects of catalpalactone on STAT1 expression via Western blotting, and the result indicated that catalpalactone concentrations exceeding 30 µM markedly inhibited STAT1 protein expression (Figure 4B). Previous studies demonstrated that *Stevia rebaudiana* suppressed NO production by inhibiting the NF-κB and IFN-β/STAT1 pathways in RAW264.7 cells [27]. In addition, Zhu et al. reported that chloride substituted 2-(2-phenethyl)-chromone (GYK-17) isolated from agarwood suppresses LPS-induced inflammatory mediator production in RAW264.7 cells by inhibiting the STAT1/3 and extracellular signal-regulated kinase 1 and 2 (ERK1/2) signaling pathways [28]. Similar to previous reports, the findings of this study demonstrate that catalpalactone inhibits LPS-induced iNOS expression by blocking IFN-β/STAT1 signaling and consequently exerts anti-inflammatory effects.

### 2.5. Effects of Catalpalactone on IRF3 and NF-κB Activation in LPS-Stimulated RAW264.7 Cells

**Figure 5:**
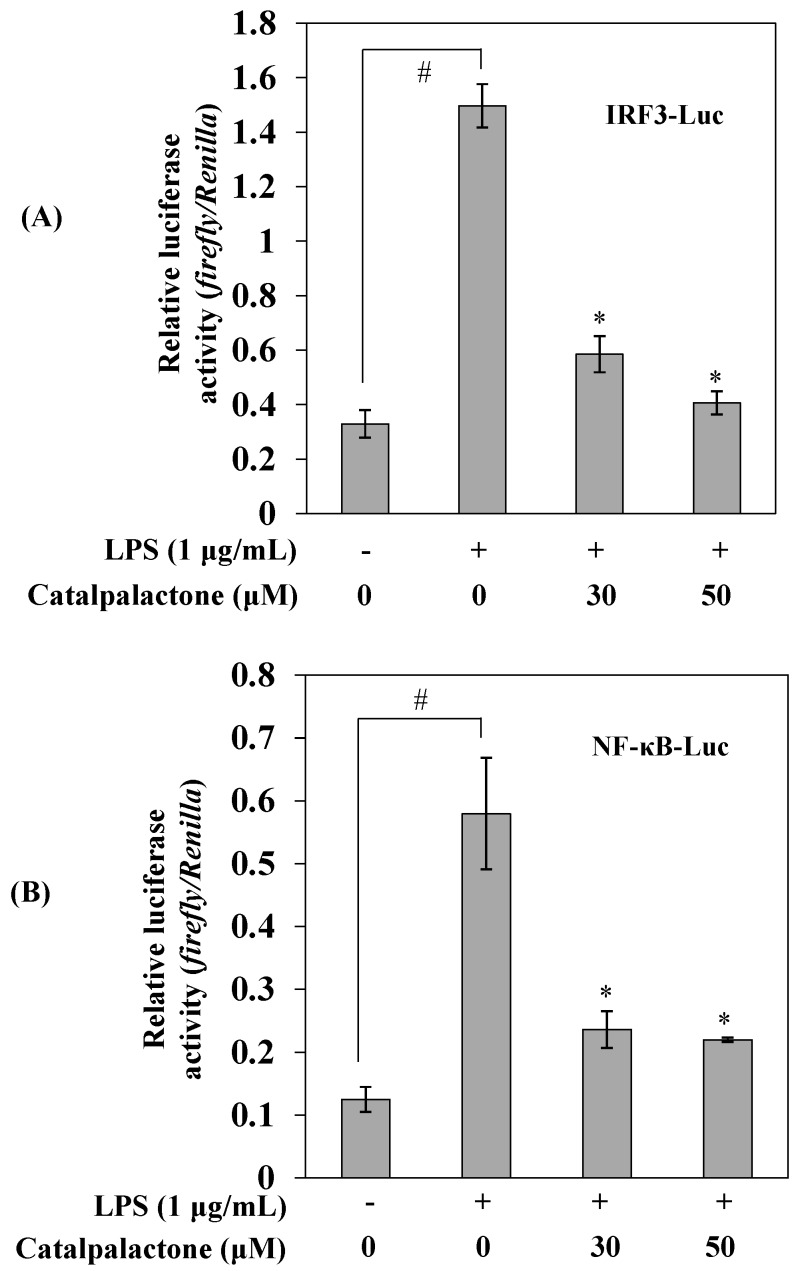
Effects of catalpalactone on interferon regulatory factor 3 (IRF3) (**A**) and nuclear factor-κB (NF-κB) (**B**) activation in LPS-stimulated RAW264.7 cells. RAW264.7 cells were transfected with pIRF3-luc and pNF-κB-luc reporter vectors. The pRL-TK vector was used as an internal control. The cells were pretreated with various concentrations of catalpalactone (30 and 50 µM) for 2 h and then stimulated with or without LPS (1 μg/mL) for 24 h. Luciferase activity was measured using a Dual-luciferase Reporter Assay System. Data are presented as the mean ± SD (*n* = 3). ^#^ *p* < 0.05 vs. control cells, * *p* < 0.05 vs. LPS-treated cells.

To further identify the mechanism by which catalpalactone exhibits anti-inflammatory effects in LPS-induced RAW264.7 cells, we performed reporter gene assays. As shown in Figure 5, stimulation with LPS significantly increased IRF3 and NF-κB reporter activity compared with that in untreated cells; however, the LPS-induced increases in reporter activity were significantly decreased in cells pretreated with catalpalactone. Lee et al. reported that luteolin exerted anti-inflammatory effects via TRIF-dependent TLR signaling by targeting TANK binding kinase 1 (TBK1) [29]. Taken together, catalpalactone blocks IRF3 and NF-κB activation and interferes with TRIF-dependent signaling, indicating that the compound inhibits the inflammatory response by suppressing the production of major inflammatory mediators.

## 3. Materials and Methods

### 3.1. Chemicals

Dulbecco’s modified Eagle’s medium (DMEM) and fetal bovine serum (FBS) were purchased from Hyclone (Logan, UT, USA). An EZ-Cytox cell viability assay kit was purchased from DAEIL Lab (Seoul, Korea). LPS, Tween 20, sodium nitrite, phenylmethylsulfonyl fluoride, NP40 cell lysis buffer, dimethyl sulfoxide, protease inhibitor cocktail, and Griess reagent were purchased from Sigma-Aldrich (St. Louis, MO, USA). An RNeasy kit was purchased from QIAGEN (Hilden, Germany). A PrimeScript 1st strand cDNA synthesis kit and SYBR premix were obtained from Takara Bio Inc. (Tokyo, Japan). Antibodies against iNOS and STAT1 were purchased from Cell Signaling Technology (Danvers, MA, USA), and an antibody against β-tubulin was purchased from Santa Cruz Biotechnology (Santa Cruz, CA, USA). Opti-MEM I medium, Lipofectamine 2000, and goat anti-rabbit and anti-mouse IgG horseradish peroxidase (HRP)-conjugated secondary antibodies were purchased from Invitrogen (Carlsbad, CA, USA). Enzyme-linked immunosorbent assay (ELISA) kits for IL-6 and TNF-α were purchased from R&D Systems (Minneapolis, MN, USA), and an ELISA kit for IFN-β was obtained from Pestka Biomedical Laboratories (Piscataway, NJ, USA).

### 3.2. Plant Materials

The wood of *C. ovata* was collected from the Medicinal Plant Garden, College of Pharmacy, Ewha Womans University in March 2014. A voucher specimen (no. EAB343) has been deposited at the Natural Product Chemistry Laboratory, College of Pharmacy, Ewha Womans University.

### 3.3. Extraction and Isolation

The dried wood of *C. ovata* (20 kg) was chopped and then extracted using MeOH (4 × 54 L) overnight at room temperature. The solvent was evaporated in vacuo to give a MeOH extract (1.5 kg), which was then suspended in distilled water (1.5 L) and sequentially partitioned with hexane (10 × 2 L), EtOAc (10 × 2 L), and BuOH (8 × 2 L). The EtOAc extract (700 g) was subjected to silica gel column chromatography (CC) using the solvent system of hexane–EtOAc (99:1 to 1:4, *v*/*v*) and EtOAc–MeOH (1:0 to 0:1, *v*/*v*) to give seven fractions (F01–F07). F05 (140 g) was applied to silica gel CC (CH_2_Cl_2_–MeOH, 1:0 to 4:1, *v*/*v*) to give nine sub-fractions (F0501–F0509) and catalpalactone (60 g) (Figure 1A). Finally, the purified catalpalactone was identified by comparing its ^1^H- and ^13^C-NMR data with published data (Supplementary Materials) [30].

### 3.4. Cell Culture

RAW264.7 macrophages were purchased from the American Type Culture Collection (Manassas, VA, USA). Cells were cultured in DMEM containing 10% FBS, 100 units/mL penicillin, and 100 μg/mL streptomycin (Invitrogen) at 37 °C in a humidified atmosphere with 5% CO_2_.

### 3.5. Cytotoxicity Assay

Cells were cultured on a 96-well plate at a density of 2 × 10^5^ cells/mL for 24 h. The cells were treated with various concentrations of catalpalactone (5, 10, 30, and 50 µM). After 24 h of incubation, cell viability was analyzed according to the manufacturer’s instructions using an EZ-Cytox cell viability assay kit. Briefly, 10 μL of the kit solution was added to each well and incubated for 4 h at 37 °C under 5% CO_2_. The cell viability was determined by measuring formazan production using an ELISA reader (Benchmark Plus, Bio-Rad, Hercules, CA, USA) based on absorbance at 480 nm. The reference wavelength was 650 nm.

### 3.6. Measurement of NO Production

Cells were cultured on a 96-well plate at a density of 2 × 10^5^ cells/mL for 24 h. The cells were treated with various concentrations of catalpalactone (5, 10, 30, and 50 µM) and incubated for 2 h. Cells were incubated with LPS (1 μg/mL) for 18 h at 37 °C under 5% CO_2_. The culture supernatant (100 μL) was reacted with Griess reagent (100 μL) in a 96-well plate and incubated at room temperature for 15 min. The absorbance was measured at 540 nm using an ELISA reader. Nitrite concentrations were calculated using a standard sodium nitrite curve. The results are presented as the mean ± SD of six replicates for one representative experiment.

### 3.7. Western Blotting

Cells were cultured on 100 mm culture dishes at a density of 2 × 10^5^ cells/mL for 24 h. The cells were treated with various concentrations of catalpalactone (5, 10, 30, and 50 µM) and incubated for 2 h. Cells were then incubated with LPS (1 μg/mL) for 18 h at 37 °C under 5% CO_2_. The cells were harvested and lysed using NP40 cell lysis buffer (containing 1 mM phenylmethylsulfonyl fluoride and 1× protease inhibitor cocktail) for 30 min on ice. The protein concentration of the cell lysate was determined using a Bio-Rad Protein Assay (Bio-Rad, Hercules, CA, USA). Aliquots of 30 μg of protein were loaded and electrophoresed on 10% SDS–polyacrylamide gels and then transferred to nitrocellulose membranes (Hybond ECL Nitrocellulose; GE Healthcare, Chandler, AZ, USA). The membranes were washed once using phosphate-buffered saline (PBS) containing 0.05% Tween 20 and blocked using PBS containing 5% skim milk and 0.05% Tween 20 for 1 h. After blocking, the membranes were incubated with target antibodies (iNOS, STAT1, and β-tubulin) at 4 °C overnight. After incubation, the membranes were washed and incubated for 2 h at room temperature with HRP-conjugated secondary antibodies diluted 1:2000 in blocking buffer. The protein bands were detected using a chemiluminescence detection system (GE Healthcare, Bucks, UK).

### 3.8. Measurements of IL-6, TNF-α, and IFN-β

Cells were cultured in a 6-well plate at a density of 2 × 10^5^ cells/mL for 24 h. The cells were pretreated with various concentrations of catalpalactone (5, 10, 30, and 50 µM) for 2 h and then stimulated with or without LPS (1 μg/mL) for 18 h. The culture supernatant was measured using ELISA kits for IL-6, TNF-α, and IFN-β according to the manufacturer’s protocol. The results are presented as the mean ± SD of three replicates for one representative experiment.

### 3.9. Luciferase Assay

Cells were cultured in a 6-well plate at a density of 2 × 10^5^ cells/mL for 24 h. pNF-κB-*Luc* (Stratagene, La Jolla, CA, USA), pIRF3-*Luc* (a kind gift from Dr. Katherine Fitzgerald, University of Massachusetts Medical School, Worcester, MA, USA), and pRL-TK control reporter vectors (Promega, Madison, WI, USA) were transfected using Lipofectamine 2000 according to the manufacturer’s protocol. After transfection, the cells were pretreated with various concentrations of 30 or 50 µM catalpalactone for 2 h and then stimulated with or without LPS (1 μg/mL) for 24 h. Luciferase activity was measured using the Dual-luciferase Reporter Assay System (Promega, Madison, WI, USA). The results are presented as the mean ± SD of three replicates for one representative experiment.

### 3.10. Quantitative Real-Time PCR Analysis

Cells were cultured in a 6-well plate at a density of 2 × 10^5^ cells/mL for 24 h. The cells were pretreated with various concentrations of catalpalactone (5, 10, 30, and 50 µM) for 2 h and then stimulated with or without LPS (1 μg/mL) for 18 h. Total RNA was isolated from the cells using an RNeasy kit (QIAGEN, Hilden, Germany). A PrimeScript 1st strand cDNA synthesis kit was used for reverse transcription (RT) according to the manufacturer’s protocol. A Chromo4 real-time PCR detection system (BioRad, Hercules, CA, USA) and SYBR Green were used for RT-PCR amplification for iNOS, IL-6, TNF-α, and β-actin under the following conditions: 50 cycles of 94 °C for 20 s, 60 °C for 20 s, and 72 °C for 30 s. The primer sequences are listed in Table 1.

The expression of target genes in comparison with β-actin was evaluated via the comparative C_T_ threshold method using the BioRad software tool Genex-Gene Expression Macro [31]. The results are presented as the mean ± SD of three replicates for one representative experiment.

### 3.11. Statistical Analysis

The differences between datasets were assessed using one-way analysis of variance followed by Tukey’s multiple comparison test. *p* < 0.05 was considered to be statistically significant.

## 4. Conclusions

We observed the anti-inflammatory activities of catalpalactone isolated from *C. ovata* in LPS-induced RAW264.7 cells. Catalpalactone was not cytotoxic in RAW264.7 cells at concentrations up to 50 µM. Moreover, our data indicated that catalpalactone inhibited NO and pro-inflammatory cytokine production and suppressed iNOS mRNA and protein expression in LPS-induced RAW264.7 cells in a concentration-dependent manner. In addition, catalpalactone suppressed STAT1 expression and IFN-β production and significantly decreased IRF3 and NF-κB activation in LPS-induced RAW264.7 cells. Taken together, catalpalactone has anti-inflammatory activity via both MyD88-dependent and independent pathway in LPS-stimulated RAW264.7 cells.

On the basis of its low cytotoxicity and potent anti-inflammatory effects, catalpalactone is suggested to be a superior medicine plant resource that can be developed to prevent and treat inflammatory diseases.

## Figures and Tables

**Table 1:** Primers used for quantitative real-time PCR.

Gene	Sequence (5′ to 3′)
iNOS	Forward: TCCTACACACCAAACTGTGTGC Reverse: CTCCAATCTCTGCCTATCCGTCTC
IL-6	Forward: GTTCTCTGGGAAATCGTGGAA Reverse: GCAAGTCCATCATCGTTGTTC
TNF-α	Forward: GCCACCACGCTCTTCTGTCTAC Reverse: GGGCTACAGGCTTGTCACTCG
β-actin	Forward: TGAGAGGGAAATCGTGCGTGAC Reverse: GCTCGTTGCCAATAGTGATGACC

## Supplementary Materials

The following are available online. Figure S1: ^1^H-NMR spectrum of catalpalactone; Figure S2: ^13^C-NMR spectrum of catalpalactone; Figure S3: HPLC analysis of catalpalactone.
