# Supplementary material for: Anti-Inflammatory Effects of Catalpalactone Isolated from Catalpa ovata in LPS-Induced RAW264.7 Cells
Source: Molecules. 2019 Mar 29;24(7):1236. doi: 10.3390/molecules24071236 (PMC6479692; doi:10.3390/molecules24071236)
Supplement: Supplementary file 1 [file molecules-24-01236-s001.pdf]

SUPPLEMENTARY DATA

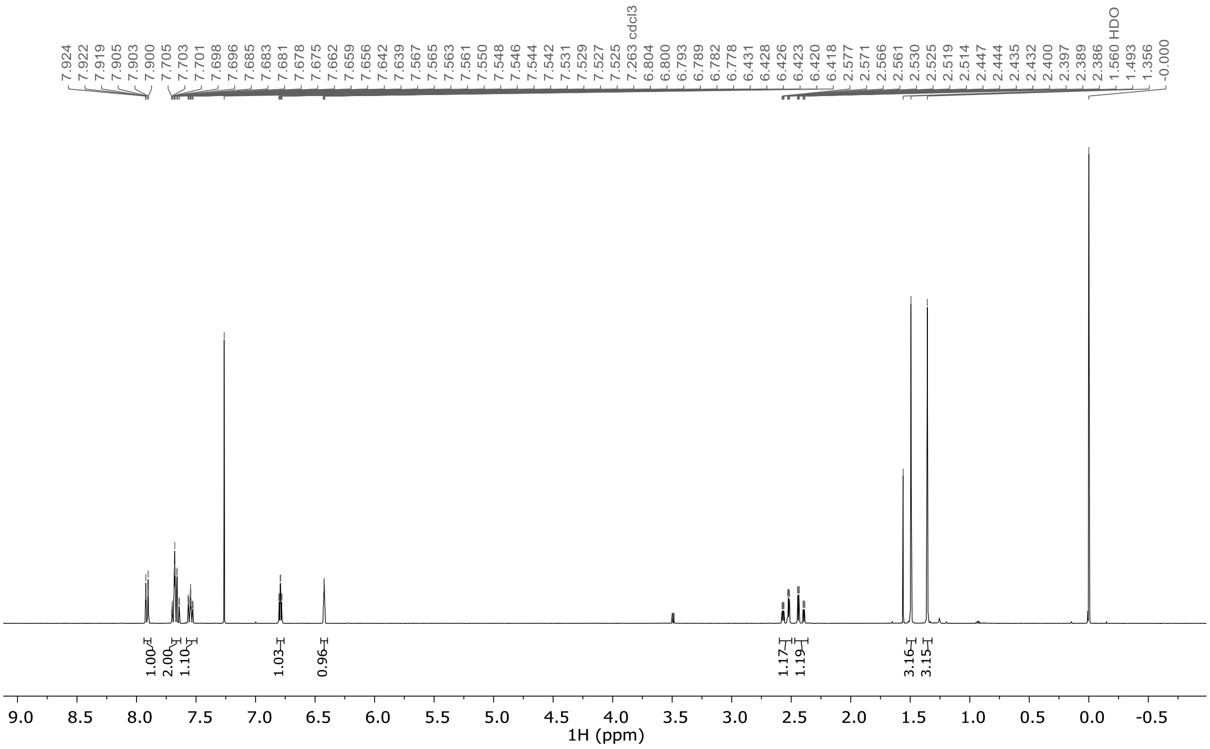

Figure S1. ^{1}H NMR spectrum (400 MHz, $CDCl_3$) of catalpalactone.

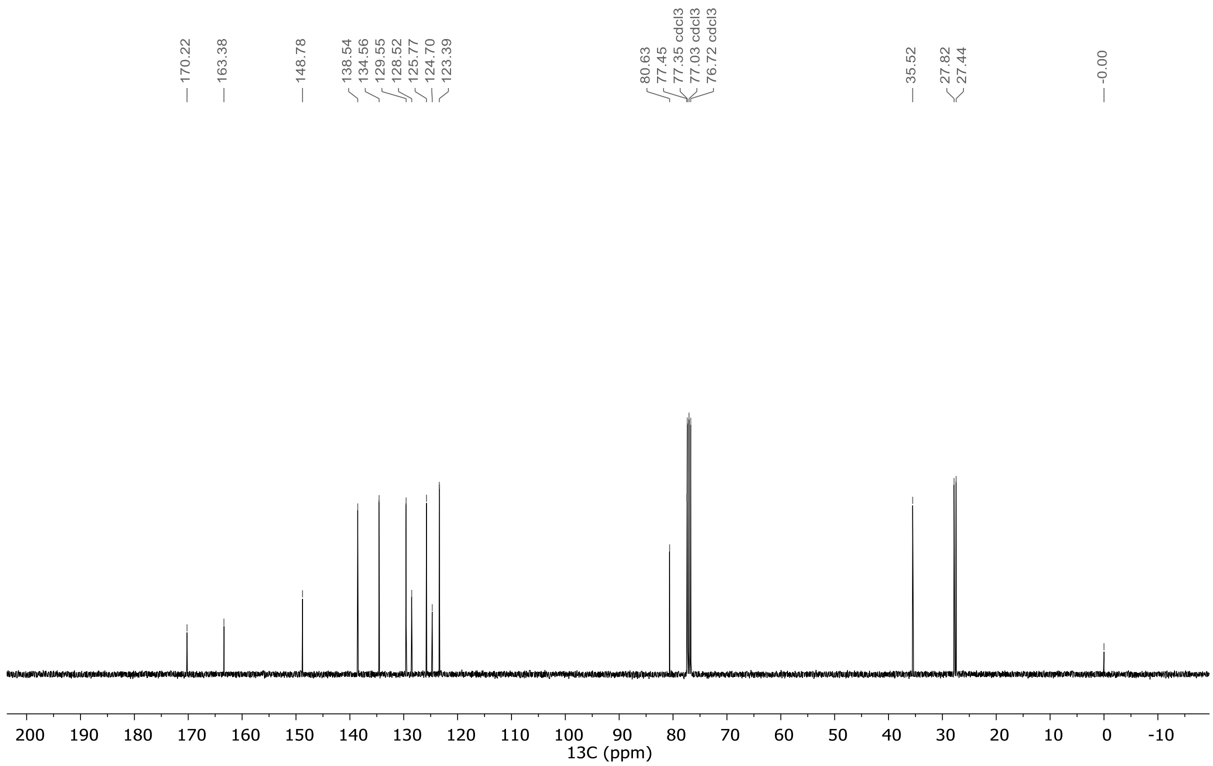

Figure S2. ^{13}C NMR spectrum (100 MHz, $CDCl_3$) of catalpalactone.

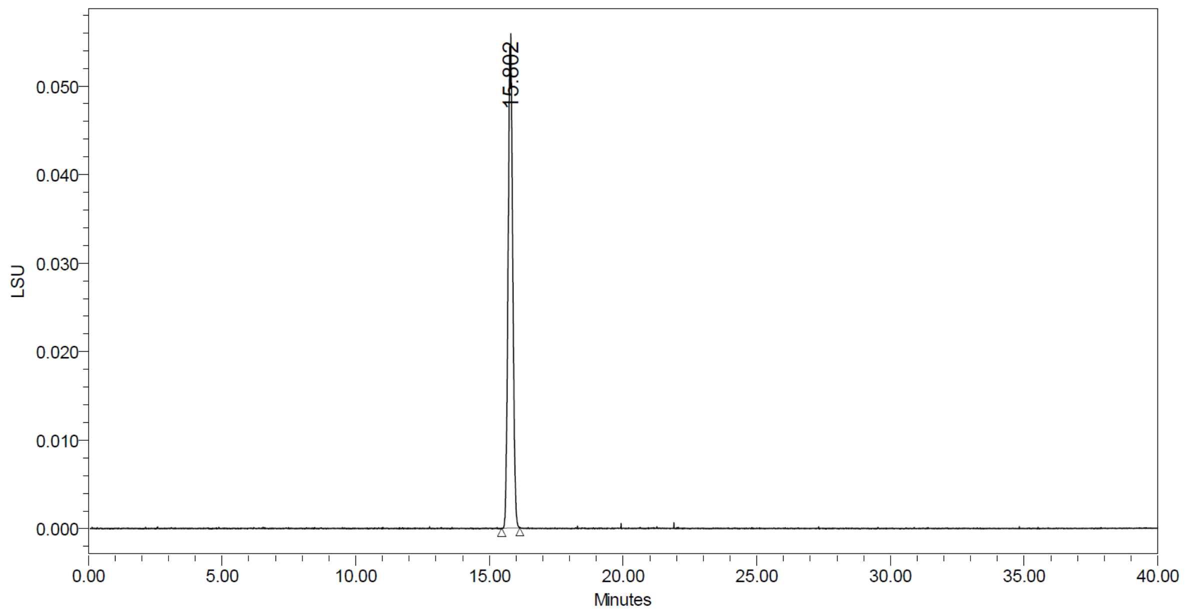

	RT	Area	% Area	Height
1	15.802	665	100.00	56

Figure S3. HPLC analysis of catalpalactone isolated from *Catalpa ovata* (0 min, MeOH–water, 2:3; 15 min, MeOH–water, 1:1; 30 min, MeOH–water, 4:1; 40 min, MeOH–water, 1:0; flow rate 1 mL/min; ELS detector). The purity is greater than 99%.
